# All policies are wrong, but some are useful—and which ones do no harm?

**DOI:** 10.1007/s11299-020-00251-3

**Published:** 2020-08-09

**Authors:** Mario Brito, Maxwell Chipulu, Ian G. Dawson, Yaniv Hanoch, Konstantinos V. Katsikopoulos

**Affiliations:** Centre for Risk Research, Department of Decision Analytics and Risk, University of Southampton Business School, Southampton, UK

**Keywords:** Covid-19, Risk, Decision, Policy

## Abstract

The five of us research and teach risk analysis with an eye towards decision support. Our work has been dedicated to taming risks and helping to make challenging decisions. But nothing had prepared us for the Covid-19 pandemic. We first had to grapple with the news coming from abroad, including, for some of us, our home countries. Then, some information and research, but mostly opinions, started coming in from our academic community, and we felt the tensions. Finally, the UK went into an unofficial and then official lockdown, and all University staff were asked to redirect their research capacity so as to support the national effort for risk analysis and decision support. As we write this on the 20th of April, many countries, including the UK, are starting to consider how to get out of lockdown. Like the previous stages of the pandemic, there is little data, perhaps a bit more research, surely many more opinions, and definitely an overwhelming amount of personal experiences and thoughts. Here we reflect on all of the above, just in case it can help the readers of this *Minds in Society* flash editorial to think and act, or at least, to not have to do so entirely on their own. As it can be expected, our collage introduces more questions than it can answer.

On March 7, one of us (YH) took the train from London to Paris. He and his loved one were planning to celebrate a birthday in style in Paris. The train was full of people, with no empty seat in sight. In Paris, the streets were booming with action and people, and the restaurants, cafes and museums were full. There were no signs that anyone was worried about the coronavirus, or that in about a week both France and the UK will be in lockdown. Sure, there were plenty of warning signs, as YH’s family in Israel was already in quarantine and Italy and Spain were reporting alarming numbers of cases and deaths, but oh well, you know. A few days after, another one of us (KK), travelled to Manchester to externally examine a PhD student. While everyone enjoyed the post-viva lunch in a grand room that used to be Alan Turing’s library, it also became clear that the UK was in for the long haul. Chris Whitty, the government’s chief medical scientist, talked about “herd immunity” and “flattening the curve”. The briefing mentioned weeks, but we all heard months. On his way back home, KK read that Greece had gone in lockdown.

The rates of infection and death were very low then. They still are low. Leading epidemiologist, research methodologist and statistician—now, that’s a good set of skills for studying Covid-19—John Ioannidis and his colleagues have calculated the death rates in the population as of April 4 (Ioannidis et al. 2020). Across 8 European countries and 4 states in the U.S., all qualifying as “hotbeds” for the pandemic, the absolute risk of dying with Covid-19 ranged from 1.7 (Germany) to 79 (New York City) per million for those under 65 years old, and from 1 in 6000 (Germany) to 1 in 420 (Spain) for those over 65 years old and no pre-existing conditions. To put those numbers into perspective, the death risk for people under 65 years old is equivalent to driving from 9 miles (Germany) to 415 miles (New York City) each day during the fatality season. And it should be emphasized that these risks refer to dying with Covid-19 but not necessarily from Covid-19.

Of course, these data mix very different periods, before lockdowns and during lockdowns. We know that social distancing must have brought the infection and death rates down, but we do not know by how much, and what other effects it had, for example on mental health and through the non-treatment of relatively minor non-coronavirus-related conditions. Such estimations would be further complicated by the initiatives of individuals, businesses and local authorities, which are not always aligned with government advice or orders. One of us (MB) comes from Portugal, where social distancing was unofficially implemented 1 week before the official lockdown. Also, MB’s friends back home did not buy into the theory of herd immunity, which tempted quite a few people in the UK, and such differences must also affect behaviour. The situation is even more vague if one asks how many people have been infected. This nobody really knows because testing is very biased, targeted at those with severe symptoms. Well, perhaps Iceland knows because they have been implementing representative, random sampling, what most countries said they would do as soon as possible. As of April 20, there were 1773 positive cases out of approximately 40,000 Icelanders tested, which is a bit over 4% (Norrestad 2020).

So, the evidence is not very clear. That’s a polite way of talking about what has been called the “evidence fiasco of the century” (Ioannidis 2020), in an opinion piece that was controversial but not because anybody responded that the available data is in fact good. Rather, whereas Ioannidis argued that we should not shut down the world, and endure all the health problems that this will ultimately cause, *without* good data; others such as Harvard epidemiologist Marc Lipsitch (2020) forcefully counterargued that, exactly *because* we do not know the facts, we ought to be conservative right now. We know which side was listened to by most governments (an exception is Sweden). It all happened so fast. How did the world move from Covid-19 risk analysis—or the absence of it—to Covid-19 decision making?

We would probably all agree that this pandemic does not represent a direct extinction threat to the human kind; world population currently increases by 200,000 people-a-day and, as of April 20, Covid-19 had killed almost 170,000 people. But this is clearly a very small consolation. We certainly all agree that we need to protect the vulnerable and the elderly. One of us (MC) has witnessed first-hand the resolve and resilience of a local community in helping a neighbor who is a brain cancer patient. Another one of us (ID) had to self-isolate because of a chest infection and surely that would make everyone think. It is *risk perception* that ultimately drives the judgment and decision making of ordinary citizens, health professionals and policy makers (Slovic 1993). Television, the newspapers and the social media appear to have played a big role in Covid-19 risk perception, and made a slow, proportionate response seem less viable.

We are of course calling for empirical research programs that would allow to comprehensively understand pandemic risk facts and perceptions across the board, in order to inform the decision models that would ultimately support policy making. We would like to close by reflecting on these pathways to policy. The title of our piece nods to modern towering statistician George Box and ancient founder of medicine Hippocrates. Box (1977) was enough of an expert on models to know that they are making wrong assumptions and wrong predictions, but they can still bring insight. The Hippocratic oath considers interventions for improving health and urges that a first standard is to *do no harm* to the patient. Modelers like us have had tons of ideas on how to mine people’s and organizations’ text narratives about risk (Fan et al. 2006), feed them into Bayesian models, multi-agent or system-dynamics simulations (Blei 2012) and find out something, anything, about what citizens, companies and societies need and how it can be provided to them by policy. Excellent academics did bring their models to completion and to the forefront with what now appears to have been an enormous impact on policy (Shipman and Wheeler 2020). As our colleagues around the world, we too are writing research proposals in order to fund consortia so that more modeling work can be done, or are just doing it anyway. This is as it should be. But we cannot help worrying that we are back to square one or, worse, that we never left it. Did scientists, policy makers and the whole society act as rationally as they should in a twenty first century crisis? Did we gather facts and uncover perceptions? Do we really know more now than we did in early March? When will we?
